# Recurrent syncope in long survivors and its association with geriatric syndromes

**DOI:** 10.1002/agm2.12240

**Published:** 2023-01-10

**Authors:** Kamal Bandhu, Akshata Rao, Ashima Nehra, Sada Nand Dwivedi, Avinash Chakrawarty, Aparajit Ballav Dey

**Affiliations:** ^1^ Department of Geriatric Medicine All India Institute of Medical Sciences New Delhi India; ^2^ Neuropsychology, Neurosciences Centre All India Institute of Medical Sciences New Delhi India; ^3^ Department of Biostatistics All India Institute of Medical Sciences New Delhi India; ^4^ Venu Geriatric Care Center New Delhi India

**Keywords:** cognitive impairment, geriatric syndrome, hearing impairment, heart disease, multimorbidity, syncope

## Abstract

**Objective:**

Syncope is a common clinical condition in the elderly, associated with significant morbidity and risk of recurrence. Recurrent syncope causing a repeated reduction in the cerebral blood flow can predispose to progressive neurodegeneration, a decline in overall health and functionality. Hence, this study was conducted to study the common causes of recurrent syncope and its association with various geriatric syndromes.

**Methodology:**

This case–control study recruited 50 cases of recurrent syncope and 50 controls, aged 75 years and older. A detailed history and sequential evaluation for aetiologies of recurrent syncope were done. Cognition, frailty, activities of daily living, depression, and nutrition were assessed using various scales.

**Results:**

Most (80%, 80/100) of the participants were males and the mean age was 80.04 ± 4.3 years. In the syncope group, 42% (21/50) of patients had arrhythmia, and 30% (15/30) had valvular heart disease. Recurrent syncope was significantly associated with lower scores on Montreal cognitive assessment scale (OR: 6.47 *P* < 0.001), four or more comorbidities (OR: 6.29 *P* < 0.001), and hearing impairment (OR: 6.21 *P* < 0.004) on multivariate logistic regression analysis.

**Conclusion:**

Recurrent syncope is significantly associated with cognitive impairment, the presence of four or more comorbidities, and hearing impairment. Conduction abnormality was the most common etiology of recurrent cardiovascular syncope. Structured evaluation and appropriate management of recurrent syncope might reduce the decline in physical, cognitive, and psychological reserve. A follow‐up longitudinal study is needed to establish this.

## Graphical abstract

Cardiovascular syncope is the most common cause of recurrent syncope in the elderly and is associated significantly with cognitive impairment, the presence of four or more comorbidities and hearing impairment. Conduction abnormality is the most common etiology for recurrent cardiovascular syncope. Structured evaluation of syncope to identify etiology and appropriate management of cardiovascular syncope might reduce the decline in physical, cognitive, and psychological reserve among older adults.

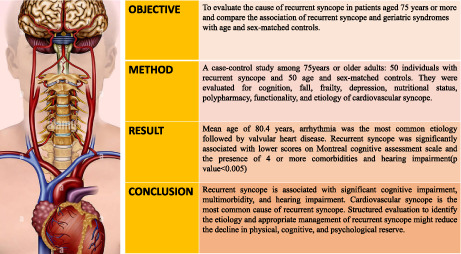

## 1 INTRODUCTION

Syncope is defined as a transient loss of consciousness due to transient global cerebral hypoperfusion characterized by rapid onset, short duration, and spontaneous complete recovery.^1^ Syncope is a common symptom in the elderly; the incidence increases rapidly with advancing age. The annual incidence of syncope is 5.7 episodes per 1000 individuals between 60 and 69 years and 11.1 episodes per 1000 individuals between 70 and 79 years, and after 80 years it rises to 19.5 per 1000 individuals.^2^ The recurrence is more frequent with increasing age, cardiac comorbidities, and the use of multiple medications.^3^ Most syncope in the elderly is due to cardiovascular etiology, most of which can be treated and further syncope prevented.^4^

The underlying mechanism for syncope is a sudden temporary reduction in cerebral perfusion mainly to the reticular activating system, which maintains consciousness.^1^ Sudden cessation of blood for 6 to 8 seconds or reduction of systolic blood pressure to 60 mmHg or less leads to transient loss of consciousness. Age‐related changes in heart rate variability, cerebral autoregulation, blood pressure, the sensitivity of baroreflex arc, intravascular volume regulation, multiple coexisting comorbidities, and concomitant medication use impair the adaptive responses and predispose the elderly to develop recurrent syncope.^5^ Cardiovascular syncope is associated with serious consequences in the elderly, but meanwhile there are therapeutic options available.^4^

Syncope is seen commonly in patients with cognitive impairment. Vascular risk factors that lead to micro and macrovascular dysregulation and age‐related changes in cerebral autoregulation, genetic susceptibility, and environmental factors play an important role in the reciprocal interaction between cerebral hypo‐perfusion and amyloid beta accumulation and neurodegeneration leading to cognitive impairment. Indeed, syncope may be the presenting feature of cognitive impairment. Neurocardiovascular instability (NCVI) or “age‐related hypotensive syndromes” are the main cause of syncope in the setting of cognitive impairment. It remains unclear whether syncope is due to a neurodegenerative process or syncope triggers an irreversible cascade, resulting in neurodegeneration and cognitive impairment.^3^, ^6^

Advancing age and comorbidities increase the risk of syncope and fall and the likelihood of being frail. The homeostenosis of various organ systems in frail elderly inhibits their response to an acute stressor like syncope and predisposes them to fall and functional decline.^3^ Recurrent syncope leads to fear of fall and depression and affects the quality of life (QoL).^7^, ^8^

Considering the above facts, the objective of our study was to evaluate the cause of recurrent syncope in patients aged 75 years or older and assess the association between recurrent syncope and geriatric syndromes and compare the same with the age‐ and sex‐matched controls without recurrent syncope.

## 2 METHODOLOGY

Recurrent syncope was defined as a discharge diagnosis of syncope (ICD‐10 R55.9) from either an admission or visit to an emergency department (hospital contact), at any time from the first diagnosis of syncope, i.e., more than one episode of syncope was considered as recurrent syncope.

In this cross‐sectional comparative study, 50 cases with recurrent syncope were recruited from the geriatric medicine ward and outpatient department of tertiary care hospital in India from March 2015 to October 2017. The Institutional Ethics Committee approved the study (IESC/T‐14/21.01.2015), and the study was conducted according to ethical guidelines established by the Declaration of Helsinki and Good Clinical Practice Guidelines. An equal number of individuals without syncope, age‐ and sex‐matched were recruited as controls. The sample size was chosen arbitrarily. Inclusion criteria for the syncope group were individuals aged 75 years or more with recurrent syncope. Inclusion criteria for the control group were individuals with no history of syncope visiting geriatric medicine OPD/ward for management of other medical illnesses. Exclusion criteria for both the groups were subjects not capable of undergoing detailed assessment and those who were previously diagnosed with cognitive impairment. After obtaining the consent for participation in the study, the participants were subjected to a detailed investigation protocol, which included a historical review of current health and coexisting comorbidities like diabetes, hypertension, chronic obstructive pulmonary disease, arthritis, self‐reported visual, hearing loss, urinary incontinence, stroke, coronary artery disease, psychiatric illness like depression and anxiety, cancer, and any other significant health issues. Further, the syncope group was evaluated sequentially for the etiology of syncope in the following order:

1. Orthostatic hypotension (when there is a reduction of ≥20 mmHg in SBP or ≥10 mmHg in DBP within 3 min of standing from a lying down position^9^) measurement and 12 lead ECG.
2. Echocardiography.
3. Holter monitoring.
4. Carotid sinus massage.
5. Head‐up tilt testing comprises two phases: (a) the supine pretilt phase; and (b) the passive head‐up tilt. The supine pre‐tilt phase lasted at least 5 min when no venous cannulation is performed and at least 20 min when venous blood sampling was done; the tilt angle of 70° for the duration of 40 min. The test was considered positive when patients experienced syncope or at least near syncope with objective modifications in heart rate (bradycardia) and/or blood pressure (hypotension) measurement.

During the sequential evaluation, when the etiology of syncope was found, the next evaluation process was not done. Though the sequential investigations approach was followed in this study, some of the patients had been investigated previously with two or more investigations and those reports were also considered in this study.

The geriatric syndromes considered in the study were: Cognitive impairment, depression, frailty, malnutrition, multimorbidity, polypharmacy, urinary incontinence, loss of functionality, fall and fear of fall, vision and hearing impairment.

Hindi Mental State Examination (HMSE)^10^ & Montreal Cognitive Assessment (MOCA)^11^ were done to screen for cognitive impairment and the scores were adjusted according to the literacy levels. For diagnosis of cognitive dysfunction, Montreal Cognitive Assessment (MOCA) was used with a score of 26 or more as normal. To better adjust the MOCA for lesser educated individuals, two points were added to the total MOCA score for those with 4 to 9 years of education and one point for 10 to 12 years of education.^12^ MOCA <26 or equivalent was suggestive of cognitive dysfunction. Depression was assessed using the Geriatric Depression Scale Short form (GDS 15 points)^13^; those with a score of 5 or more were considered positive for depression. Frailty was diagnosed by calculation of frailty index (≥0.25) using 36 deficit model Rockwood criteria.^14^ Nutritional status was assessed using the Mini Nutritional Assessment Short Form (MNA‐SF) and were classified as malnourished (0–7), at risk of malnutrition (8–11), and normal (12–14) based on the total score.^15^ Assessment of functionality concerning instrumental and basic activities of daily living was done in both the groups using Lawton's scale^16^ and Barthel's index,^17^ respectively. Subjects with a score of 8/8 and 20/20 on Lawton's scale and Barthel's index, respectively, were considered functionally independent. Fall assessment was done by asking a single question “Have you experienced a fall in the last year?” and any history of fear of fall was also asked.

### 2.1 Statistical analysis

Data were managed in an MS‐Excel database and analyzed using STATA 14 software.^18^ The correlation of syncope and demographic characters was done. Descriptive analysis was done for all variables and frequency (percentage) for qualitative and mean ± SD or median (quartiles, q1 & q3) were calculated for quantitative variables. The categorical variables were analyzed for association using the Chi‐square test. *p* value < 0.05 was considered significant. To measure the strength of association, univariate logistic regression analysis was done with syncope as dependent variable and was expressed as odds ratio with 95% confidence interval. The covariates that were significantly associated were further analyzed with multivariate logistic regression.

## 3 RESULTS

In this cross‐sectional study, with a sample size of 100, 50 individuals with recurrent cardiovascular syncope patients and 50 age‐ and sex‐matched controls were studied. The mean age of the patient enrolled was 80.4 (±4.30) years in both syncope case and non‐syncope control groups of which 80% were male. Type 2 diabetes and hypertension were the two most common comorbidities in both groups. The frequency of coronary artery disease was significantly higher in the recurrent syncope group. All the patients in the syncope group had multimorbidity (two or more comorbidities). Recurrent syncope was significantly associated with four or more comorbidities (76% vs. 40% *p* < 0.001). The baseline characteristics and comorbidities of the study population are depicted below in Table 1.

**TABLE 1:** Baseline characteristics and comorbidities in the syncope and non‐syncope group.

Characteristics	Syncope (*n* = 50)	Non‐syncope (*n* = 50)	*P* Value
Age (mean ± SD)	80.04 ± 4.3	80.04 ± 4.3	1.000**
Males	40 (80%)	40 (80%)	1.000
Females	10 (20%)	10 (20%)	
Literacy	41 (82%)	43 (86%)	0.707
Average BMI (kg/m^2^)	22.39	22.74	
Smoking	26 (52%)	20 (40%)	0.229
Alcohol	18 (36%)	12 (24%)	0.19
Hypertension	34 (68%)	36 (72%)	0.663
Diabetes	22 (44%)	13 (26%)	0.059
Joint pain	28 (56%)	33 (66%)	0.400
Dizziness	48 (96%)	11 (22%)	**<0.001**
Vertigo	3 (6%)	9 (18%)	0.121*
Anemia	20 (40%)	17 (34%)	0.534
Hypothyroidism	2 (4%)	4 (8%)	0.059*
BPH	17 (34%)	20 (40%)	0.534
Constipation	19 (38%)	22 (44%)	0.542
COPD	10 (20%)	13 (26%)	0.476
CKD	6 (12%)	3 (6%)	0.295
CAD	25 (50%)	14 (28%)	**0.024**
Cancer	4 (8%)	1 (2%)	0.362*
Comorbidities			
≤1	0 (0%)	4 (8%)	0.117*
≥2	50 (100%)	46 (92%)	0.117*
≥4	38 (76%)	20 (40%)	**<0.001**

*Note*: *P* Value calculated using Chi‐square, *Fischer exact test, **unpaired T‐test. Bold indicates significantly different in syncope and non syncope group.

Abbreviations: BPH, Benign prostatic hyperplasia; CAD, Coronary artery disease; CKD, Chronic kidney disease; COPD, Chronic obstructive pulmonary disease.

When the subjects were evaluated according to the sequential protocol for evaluating the etiology of syncope, the most common cause of recurrent syncope was cardiovascular syncope, among which arrhythmia was the most common cause as seen in 42% of the subjects. Sick sinus syndrome was seen in 6% of the subjects with syncope and sinus bradycardia was seen in 2% of the subjects. Valvular heart disease was the second most common etiology of cardiovascular syncope in the elderly accounting for 30% of the cases. Aortic stenosis was the most common valvular disease and accounted for 66.66% of the valvular heart disease causing syncope. DCMP and OH were seen among 10% and 4% of syncope subjects, respectively. Of the syncope patients, 14% (7/50) were found to have other multiple causes of syncope. Cases with multifactorial etiology include AF and a combination of others mentioned in Table 2. All 50 patients underwent 12 lead ECG and blood pressure assessment for orthostatic hypotension, 26 patients underwent echocardiography, 6 patients underwent Holter testing, and 1 patient underwent carotid sinus massage and head up tilt test.

**TABLE 2:** Etiology of recurrent syncope

Etiology	Number of syncope patients (n = 50) (%)
OH	2 (4%)
Arrhythmia	**21 (42%)**
a. CHB & 2nd‐degree block	17
b. Sinus bradycardia	1
c. Sick sinus syndrome	3
Valvular heart disease	**15 (30%)**
a. Aortic stenosis	10
b. Other valvular disease	5
DCMP	5 (10%)
Multifactorial	7 (14%)

*Note*: Bold indicates the 2 most common causes of recurrent cardiovascular syncope.

Cognitive assessment using MOCA and HMSE showed significant impairment in the recurrent syncope group as compared to non‐syncope subjects with a *p* value of 0.001 and 0.002, respectively. Similarly, frailty and hearing impairment were also significantly associated with recurrent syncope, with a *p* value of 0.017 and 0.019, respectively. About 34% of syncope subjects were either malnourished or at risk of malnutrition as assessed using the Mini Nutritional Assessment short form scale for nutrition. Polypharmacy was seen in more than two‐thirds of the study population (Table 3).

**TABLE 3:** Comparison of various geriatric syndrome assessments between syncope and non‐syncope group

Characteristics	Syncope (*n* = 50) (%)	Non‐syncope (*n* = 50) (%)	*P* Value
MOCA impaired	33 (66)	16 (32)	**0.001**
HMSE impaired	28 (56)	13 (26)	**0.002**
Frailty	40 (80)	29 (58)	**0.017**
BADL impaired	33 (66)	25 (50)	0.073
IADL impaired	38 (74)	31 (62)	0.089
GDS short form (≥5)	31 (62)	22 (44)	0.071
Fall	12 (24)	5 (10)	0.062
Fear of fall	17 (34)	9 (18)	0.068
Urinary incontinence	22 (44)	22 (44)	1.000
MNA‐SF			
Malnourished (0–7)	2 (4)	0 (0)	0.261
At the risk of malnutrition (8–11)	15 (30)	12 (24)	
Normal (12–14)	33 (66)	38 (76)	
Polypharmacy (≥5 drugs)	40 (80)	36 (72)	0.349
Vision impairment	26 (52)	34 (68)	0.102
Hearing impairment	43 (86)	33 (66)	**0.019**

*Note*: *P* Value calculated using Chi‐square. Bold indicates that there was significant difference between syncope and non syncope group.

Univariate analysis with syncope as dependent variable showed there was significant association with coronary artery disease, cognitive impairment, hearing impairment, frailty, and presence of four or more comorbidities. On further multivariate analysis, significant association of syncope was only observed with cognitive impairment, hearing impairment, and presence of four or more comorbidities (Table 4).

**TABLE 4:** Univariate and multivariate regression analysis of various covariates with syncope

Variables	Univariate analysis		Multivariate analysis	
	OR (95% CI)	*P* Value	OR (95% CI)	*P* Value
Age	1.0 (0.91–1.09)	1.000		
Sex	1 (reference)			
	1.0 (0.37–2.66)	1.000		
Comorbidities				
CAD	2.57 (1.12–5.89)	**0.026**	1.69 (0.61–4.67)	0.313
HTN	0.83 (0.35–1.95)	0.663		
DM	2.24 (0.96–5.19)	0.061		
CKD	2.13 (0.50–9.07)	0.303		
Arthritis	0.71 (0.32–1.60)	0.411		
Anemia	1.29 (0.57–2.92)	0.535		
COPD	0.71 (0.28–1.82)	0.477		
Cancer	4.26 (0.46–39.54)	0.202		
Habits				
Smoking	1.78 (0.75–4.24)	0.193		
Alcohol	1.78 (0.75–4.25)	0.193		
Geriatric syndromes				
BADL	1 (reference)			
	1.94 (0.87–4.34)	0.107		
IADL	1 (reference)			
	1.94 (0.82–4.61)	0.133		
MoCA	1 (reference)			
	4.12 (1.79–9.49)	**0.001**	6.47 (2.21–18.91)	**0.001**
MNA‐SF	1(reference)			
	1.63 (0.68–3.91)	0.272		
Vision	0.51 (0.23–1.15)	0.104		
Hearing	3.16 (1.18–8.52)	**0.023**	6.21 (1.79–21.53)	**0.004**
Urinary incontinence	1.00 (0.45–2.20)	1.00		
Polypharmacy	1.55 (0.61–3.93)	0.351		
Frailty	2.89 (1.18–7.07)	**0.019**	1.54 (0.52–4.56)	0.440
Constipation	0.78 (0.35–1.73)	0.542		
GDS	1 (reference)			
	2.07 (0.93–4.61)	0.073		
Comorbidities	1 (reference)			
	4.75 (2.01–11.24)	**<0.001**	6.29 (2.08–18.99)	**0.001**
Falls	2.84 (0.92–8.79)	0.070		
Fear of falls	2.35 (0.93–5.94)	0.072		

*Note*: Bold indicates Significant in univariate and multivariate analysis.

## 4 DISCUSSION

In this study, we evaluated the etiology of recurrent syncope in the very old (75 years and older) and its association with various geriatric syndromes. The mean age of the population being 80 years formed a relatively old cohort for the Indian standard of life expectancy, which is 69 years.^19^ Finding the etiology of syncope in the elderly is challenging as the events are frequently unwitnessed and the patients are less likely to have prodromal symptoms, may have amnesia for loss of consciousness, and it could be multifactorial. The prevalence of cardiac disease (arrhythmias and structural heart diseases) rises dramatically with age and hence the incidence of cardiac syncope also increases with age.^20^ Cardiac rhythm disturbance is the most common cause of cardiovascular syncope and is responsible for approximately 20% of syncopal episodes in the elderly.^21^ Also in this study, arrhythmias were found to be the most common etiology followed by valvular heart disease. Aortic stenosis predominated valvular heart disease. As age advances, the incidence of degenerative valvular lesions increases. Epidemiological studies have determined that more than one in eight people aged 75 and older have moderate or severe aortic stenosis (AS). Presyncope and syncope are common symptoms of AS and are also associated with the severity of aortic stenosis. The prevalence of moderate AS is to be consistently estimated at ~2% among individuals aged 70 to 80 years, with the prevalence increasing to 3% to 9% after the age of 80 years. In the US population‐based series by Nkomo and colleagues, the prevalence of AS was strongly associated with age, with an odds ratio of 2.5 (95% CI 2.0–3.1) per decade of increasing age.^22^ Coronary artery disease is known to be associated with aortic stenosis as well as myocardial infarction and is also seen to be associated with syncope.^23^ In this study, we found a significant association between coronary artery disease and syncope as compared with non‐syncope subjects.

In our study, we found that individuals with recurrent syncope were four times more likely to have cognitive impairment. Older adults have a diminished cerebrovascular reserve and autoregulation and the occurrence of syncope in them results in a further decrease in cerebral blood flow resulting in unrelenting cerebral hypoperfusion. When this cerebral hypoperfusion is below a certain threshold, i.e., critically attained threshold of cerebral hypoperfusion (CATCH), it results in changes in brain microvasculature and causes metabolic energy crisis leading to ischemic injury of neurons, which results in progressive neurodegenerative and atrophic changes in the brain. This metabolic crisis accelerates oxidative stress and pro‐inflammatory mechanisms, which results in increased amyloid beta deposition, aberrant protein synthesis, and disrupts synaptic plasticity leading to cognitive decline in the patient. In animal models, transient hypotensive episodes following bilateral carotid occlusion reduce synaptic plasticity and exacerbate both cognitive and motor dysfunction. Intriguingly, the cognitive deficits after permanent carotid occlusion are associated with elevated amyloid beta in the brain.^4^, ^24^, ^25^, ^26^ Frewen et al. studied the association of syncope and cognitive impairment in The Irish longitudinal study on aging (TILDA) and found that those participants with syncope and or non‐accidental falls in the previous year had a poor global cognitive performance in comparison to the controls.^27^

The prevalence of hearing impairment was 76% in our study population (86% in syncope group, 66% in non‐syncope group). As observed previously, the prevalence of hearing impairment increases with age. Sharma et al.^28^ found a prevalence of 81.4% in patients aged 80 years or older. Hearing impairment was significantly higher in the recurrent syncope group. This may be because of the reduced vascular supply to the inner ear. The cochlea is supplied by labyrinthine artery, which is an end branch of the vertebrobasilar system and hence it is sensitive to any restrictions in local and systemic circulation. Common systemic cardiovascular diseases are observed to be associated with hearing loss especially in elderly patients. It has been observed that patients with hearing loss of unknown etiology have been found eight times more prone to have concomitant ischemic heart disease than their healthy peers. It has been studied that 60% decrease in cerebral blood flow is known to cause TTS (temporary threshold shift) by means of cochlear hypoxia. Certain commonly used cardiovascular drugs like diuretics and beta blockers are associated with hearing loss as well.^29^, ^30^

Multimorbidity, defined as the presence of two or more comorbid conditions, is a growing public health challenge, particularly in the elderly. In our study, multimorbidity was present in 100% and 92% of the participants in the syncope and non‐syncope group. Previous studies have reported an overall prevalence of multimorbidity between 24% and 83%.^31^, ^32^ On further analysis, a higher number of comorbidities was significantly associated with recurrent syncope in our study. With advancing age, there are blunted adaptive responses, contracted blood volume, age‐related diastolic dysfunction, and other organ‐related homeostenosis, and an increasing number of comorbidities increases the likelihood of being frail and polypharmacy. Polypharmacy was observed in 80% of subjects with syncope as compared to 72% in subjects without syncope though it was not statistically significant. Age‐related physiological changes, comorbidities, and concomitant medication use also increase the risk of syncope.^3^ In our study, multimorbidity, frailty and polypharmacy were also higher in the recurrent syncope group, but statistically significant association was found only with presence of four or more comorbidities on multivariate analysis. Syncope most often presents with a history of fall. Syncope has been seen to be strongly associated with fear of fall (FoF) as well. In our study, fall and FoF were found to be more in the recurrent syncope subjects, though not statistically significant. This may be because, as observed previously, only about 20% of cardiovascular syncope in patients older than 70 appears as a fall.^33^ As cardiovascular syncope was the most common etiology in our study, it might have been a reason for majority of recurrent syncope group to experience a transient LOC but not experience a fall.

This study has strengths and limitations like any other cross‐sectional study. We have enrolled patients from a tertiary care center, which caters to and represents a heterogeneous population. Both outpatient and inpatients were included in the study, the geriatric syndrome prevalence may be higher in inpatients, but as inpatients were also included in the control group, there is no significant difference. Due to limited resources and time constraints, sequential evaluation was chosen for this study; multifactorial causation might be missed due to this approach. The sample size of the study was small; thus, the study findings cannot be well extrapolated to the general population. This study is a cross‐sectional study, hence a longitudinal study should be done to establish the causal relationship between the syncope and decline in physiological reserve.

## 5 CONCLUSION

Recurrent syncope is strongly associated with cognitive impairment, presence of four or more comorbidities, and hearing impairment. Conduction abnormality was the most common etiology of recurrent cardiovascular syncope, which is treatable, thus preventing further syncopal episodes in older adults. Structured evaluation of syncope to identify the etiology of syncope and appropriate management of cardiovascular syncope might reduce the decline in physical, cognitive, and psychological reserve.

## AUTHOR CONTRIBUTIONS

Kamal Bandhu is the Principal Investigator of this study. Dr Aparajit Ballav Dey designed the study and Akshata Rao and Sada Nand Dwivedi analyzed the data. Akshata Rao and Kamal Bandhu wrote the initial draft of the manuscript. Dr Dey, Ashima Nehra, and Avinash Chakrawarty critically revised the manuscript. All authors had responsibility for accuracy of the final content and read and approved the final manuscript.

## FUNDING INFORMATION

This work was supported by the Department of Geriatric Medicine, All India Institute of Medical Sciences, New Delhi as the post‐graduate dissertation of Dr. Kamal Bandhu.

## CONFLICT OF INTEREST

The author declares that the research was conducted in the absence of any commercial or financial relationships that could be construed as a potential conflict of interest.

## ETHICAL APPROVAL

Ethical approval was obtained from the Institutional Ethics Committee (IESC/T‐14/21.01.2015), and the study was conducted according to ethical guidelines established by the Declaration of Helsinki and Good Clinical Practice Guidelines. Informed consent was obtained from all participants.

## References

[1] Moya A , Sutton R , Ammirati F , et al. Guidelines for the diagnosis and management of syncope (version 2009). Eur Heart J. 2009;30(21):2631‐2671. doi:10.1093/eurheartj/ehp298 19713422 PMC3295536

[2] Soteriades ES , Evans JC , Larson MG , et al. Incidence and prognosis of syncope. N Engl J Med. 2002;347(12):878‐885. doi:10.1056/NEJMoa012407 12239256

[3] Wong CW . Complexity of syncope in elderly people: a comprehensive geriatric approach. Hong Kong Med J Xianggang Yi Xue Za Zhi. 2018;24(2):182‐190. doi:10.12809/hkmj176945 29658484

[4] Dey AB , Stout NR , Kenny RA . Cardiovascular syncope is the most common cause of drop attacks in the elderly. Pacing Clin Electrophysiol. 1997;20(3):818‐819. doi:10.1111/j.1540-8159.1997.tb03911.x 9080517

[5] Collins O , Kenny RA . Association between syncope and cognitive impairment. Aging Health. 2011;7(1):143‐153. doi:10.2217/ahe.11.1

[6] Maule S , Caserta M , Bertello C , et al. Cognitive decline and low blood pressure: the other side of the coin. Clin Exp Hypertens. 2008;30(8):711‐719. doi:10.1080/10641960802573344 19021022

[7] van Dijk N , Sprangers MA , Colman N , Boer KR , Wieling W , Linzer M . Clinical factors associated with quality of life in patients with transient loss of consciousness. J Cardiovasc Electrophysiol. 2006;17(9):998‐1003. doi:10.1111/j.1540-8167.2006.00533.x 16764705

[8] Bhangu JS , King‐Kallimanis B , Cunningham C , Kenny RA . The relationship between syncope, depression and anti‐depressant use in older adults. Age Ageing. 2014;43(4):502‐509. doi:10.1093/ageing/afu003 24496179

[9] Ringer M , Lappin SL . Orthostatic Hypotension. StatPearls Publishing; 2021. Accessed June 22, 2022. https://www.ncbi.nlm.nih.gov/books/NBK448192/ 28846238

[10] Ganguli M , Ratcliff G , Chandra V , et al. A hindi version of the MMSE: the development of a cognitive screening instrument for a largely illiterate rural elderly population in India. Int J Geriatr Psychiatry. 1995;10(5):367‐377. doi:10.1002/gps.930100505

[11] Nasreddine ZS , Phillips NA , Bédirian V , et al. The Montreal cognitive assessment, MoCA: a brief screening tool for mild cognitive impairment. J Am Geriatr Soc. 2005;53(4):695‐699. doi:10.1111/j.1532-5415.2005.53221.x 15817019

[12] Chertkow H , Nasreddine Z , Johns E , Phillips N , McHenry C . P1‐143: the Montreal cognitive assessment (MoCA): validation of alternate forms and new recommendations for education corrections. Alzheimers Dement. 2011;7(4S_Part_5):S157. doi:10.1016/j.jalz.2011.05.423

[13] Brown LM , Schinka JA . Development and initial validation of a 15‐item informant version of the geriatric depression scale. Int J Geriatr Psychiatry. 2005;20(10):911‐918. doi:10.1002/gps.1375 16163741

[14] Blodgett JM , Theou O , Howlett SE , Rockwood K . A frailty index from common clinical and laboratory tests predicts increased risk of death across the life course. GeroScience. 2017;39(4):447‐455. doi:10.1007/s11357-017-9993-7 28866737 PMC5636769

[15] Kaiser MJ , Bauer JM , Ramsch C , et al. Validation of the mini nutritional assessment short‐form (MNA‐SF): a practical tool for identification of nutritional status. J Nutr Health Aging. 2009;13(9):782‐788. doi:10.1007/s12603-009-0214-7 19812868

[16] Lawton MP , Brody EM . Assessment of older people: self‐maintaining and instrumental activities of daily living. Gerontologist. 1969;9(3 Part 1):179‐186. doi:10.1093/geront/9.3_Part_1.179 5349366

[17] Collin C , Wade DT , Davies S , Horne V . The Barthel ADL index: a reliability study. Int Disabil Stud. 1988;10(2):61‐63. doi:10.3109/09638288809164103 3403500

[18] StataCorp . Stata Statistical Software: Release 14. StataCorp LP; 2015.

[19] Average Life Expectancy. Accessed June 18, 2022. https://pib.gov.in/pib.gov.in/Pressreleaseshare.aspx?PRID=1606209

[20] O'Brien H , Anne Kenny R . Syncope in the elderly. Eur Cardiol Rev. 2014;9(1):28‐36. doi:10.15420/ecr.2014.9.1.28 PMC6159456 30310482

[21] McIntosh S , Da Costa D , Kenny RA . Outcome of an integrated approach to the investigation of dizziness, falls and syncope in elderly patients referred to a “syncope” clinic. Age Ageing. 1993;22(1):53‐58. doi:10.1093/ageing/22.1.53 8438668

[22] Nkomo VT , Gardin JM , Skelton TN , Gottdiener JS , Scott CG , Enriquez‐Sarano M . Burden of valvular heart diseases: a population‐based study. Lancet Lond Engl. 2006;368(9540):1005‐1011. doi:10.1016/S0140-6736(06)69208-8 16980116

[23] Iung B , Vahanian A . Epidemiology of valvular heart disease in the adult. Nat Rev Cardiol. 2011;8(3):162‐172. doi:10.1038/nrcardio.2010.202 21263455

[24] de la Torre JC . Pathophysiology of neuronal energy crisis in Alzheimer's disease. Neurodegener Dis. 2008;5(3–4):126‐132. doi:10.1159/000113681 18322369

[25] Lunn S , Crawley F , Harrison MJ , Brown MM , Newman SP . Impact of carotid endarterectomy upon cognitive functioning. A systematic review of the literature. Cerebrovasc Dis Basel Switz. 1999;9(2):74‐81. doi:10.1159/000015901 9973649

[26] De Jong GI , De Vos RAI , ENHJ S , PGM L . Cerebrovascular hypoperfusion: a risk factor for Alzheimer's disease? Ann N Y Acad Sci. 1997;826(1):56‐74. doi:10.1111/j.1749-6632.1997.tb48461.x 9329681

[27] Frewen J , King‐Kallimanis B , Boyle G , Kenny RA . Recent syncope and unexplained falls are associated with poor cognitive performance. Age Ageing. 2015;44(2):282‐286. doi:10.1093/ageing/afu191 25520310

[28] Sharma RK , Lalwani AK , Golub JS . Prevalence and severity of hearing loss in the older old population. JAMA Otolaryngol Neck Surg. 2020;146(8):762‐763. doi:10.1001/jamaoto.2020.0900 PMC7317651 32584401

[29] Lim DP , Stephens SDG . Clinical investigation of hearing loss in the elderly*. Clin Otolaryngol Allied Sci. 1991;16(3):288‐293. doi:10.1111/j.1365-2273.1991.tb02054.x 1879075

[30] Yildirim N . HearingImpairment in VascularDisorders. Van Tıp Derg. 2012;19(3):149‐157.

[31] Kshatri JS , Palo SK , Bhoi T , Barik SR , Pati S . Prevalence and patterns of multimorbidity among rural elderly: findings of the AHSETS study. Front Public Health. 2020;8:582663. doi:10.3389/fpubh.2020.582663 33251177 PMC7676903

[32] Pati S , Swain S , Hussain MA , et al. Prevalence and outcomes of multimorbidity in South Asia: a systematic review. BMJ Open. 2015;5(10):e007235. doi:10.1136/bmjopen-2014-007235 PMC4606435 26446164

[33] Ungar A , Rafanelli M , Iacomelli I , et al. Fall prevention in the elderly. Clin Cases Miner Bone Metab. 2013;10(2):91‐95. 24133524 PMC3797008

